# Novel Thiochroman‐4‐one Derivatives Bearing a Thiosemicarbazide Scaffold With Potential Antioxidant, α‐Glucosidase Inhibitory, and Antibacterial Activities

**DOI:** 10.1002/cbdv.71927

**Published:** 2026-11-14

**Authors:** Pei Li, Wenmin Pan, You Zhang, Xiang Wang

**Affiliations:** ^1^ College of Life and Health Science Kaili University Kaili China; ^2^ Guizhou Key Laboratory of Miao Medicine Qiandongnan Engineering and Technology Research Center for Comprehensive Utilization of National Medicine Kaili University Kaili China

**Keywords:** antibacterial activity, antioxidant activity, thiochroman‐4‐one, thiosemicarbazide, α‐glucosidase inhibitory activity

## Abstract

In this work, a series of novel thiochroman‐4‐one derivatives incorporating a thiosemicarbazide moiety were rationally designed and synthesized. In vitro bioactivity assays demonstrated that most target compounds exerted potent ABTS radical scavenging and α‐glucosidase inhibitory activities. Notably, compound **6m** exhibited superior bioactivities, with an IC_50_ value of 12.96 µM for ABTS radical scavenging and 27.48 µM for α‐glucosidase inhibition, which were markedly stronger than those of the positive controls Trolox and acarbose, respectively. Molecular docking and 100 ns molecular dynamics (MD) simulation results suggested that compound **6m** may form plausible and stable non‑covalent interactions with the active sites of human peroxiredoxin 5 (PDB ID: 4K7N) and human α‑glucosidase (PDB ID: 3TOP). In silico druggability and ADMET evaluations suggested that compound **6m** might possess favorable physicochemical properties and biosafety profiles; these computational predictions remain to be validated by further biological experiments. In addition, compound **6k** showed the highest antibacterial potency, with EC_50_ values of 39.37 µM against *Xoo* and 49.21 µM against *Xac*, which were higher than bismerthiazol and thiodiazole copper.

## Graphical abstract

Compound **6m** displayed superior ABTS scavenging, α‐glucosidase inhibitory, and antibacterial activities, which were stronger than the positive controls. Molecular docking and MD simulations revealed stable interactions between compound **6m** and the active site of human peroxiredoxin 5 (PDB ID: 4K7N) and human α‐glucosidase (PDB ID: 3TOP).

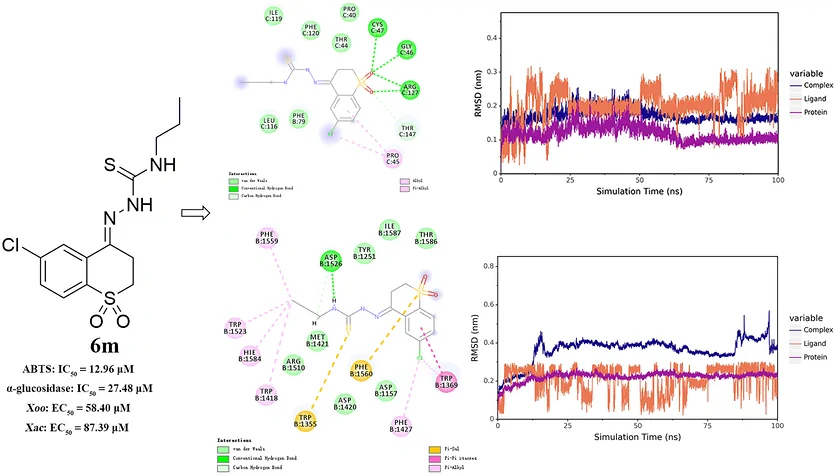

## 1 Introduction

Oxidative stress is a critical factor contributing to insulin resistance and pancreatic islet damage [1]. Excessive accumulation of reactive oxygen species (ROS) impairs cellular functions, triggers inflammatory responses, and accelerates the progression of diabetic complications [2]. Antioxidant compounds with strong radical scavenging capacity can neutralize ROS, protect cells from oxidative damage, improve insulin sensitivity, and maintain glucose homeostasis [3]. Considering the synergistic relationship between postprandial hyperglycemia and oxidative stress, dual‐functional agents combining α‐glucosidase inhibition and antioxidant activity are more advantageous than single‐target drugs [4]. Such dual‐activity molecules are capable of lowering blood glucose and alleviating oxidative injury simultaneously, thus providing comprehensive protection against metabolic disorders and their complications. Therefore, they have attracted extensive research attention in the field of medicinal chemistry.

α‐Glucosidase is a key carbohydrate‐hydrolyzing enzyme located on the brush border of the small intestine. It catalyzes the hydrolysis of complex carbohydrates into absorbable monosaccharides and directly determines the elevation rate of postprandial blood glucose [5]. Inhibition of α‐glucosidase can effectively delay the digestion and absorption of carbohydrates, thereby reducing postprandial blood glucose levels without stimulating insulin secretion. This strategy has been clinically validated for the treatment of type 2 diabetes mellitus (T2DM) [6]. However, clinically applied α‐glucosidase inhibitors such as acarbose have obvious limitations, including moderate inhibitory potency, severe gastrointestinal side effects, and a lack of antioxidant activity [7]. These drawbacks restrict their long‐term application in preventing diabetic complications, creating an urgent demand for novel potent and multifunctional candidate drugs.

Beyond pharmaceutical applications, plant bacterial diseases caused by *Xanthomonas* species have become a major threat to global agricultural production, leading to severe yield reduction and quality deterioration of crops including rice and citrus [8]. *Xanthomonas oryzae* pv. *oryzae* (*Xoo*) and *Xanthomonas axonopodis* pv. *citri* (*Xac*) are two representative phytopathogens, which cause rice bacterial blight and citrus canker, respectively [9]. Traditional agrochemicals such as bismerthiazol and thiodiazole copper are gradually losing efficacy due to the emergence of drug resistance, unsatisfactory field performance, and potential environmental risks [10]. Hence, it is imperative to develop highly efficient, eco‐friendly, and structurally novel antibacterial agents for sustainable plant disease management.

Previous studies have confirmed that sulfur‐containing heterocyclic compounds and thiosemicarbazide conjugates possess promising antibacterial activity against phytopathogens [11, 12]. In this context, constructing a single molecular skeleton with both hypoglycemic and antibacterial activities is expected to obtain multifunctional compounds with dual values for pharmaceutical and agrochemical development. Thiochroman‐4‐one is a privileged sulfur‐containing heterocyclic scaffold with flexible structural modification potential and diverse biological functions, such as antioxidant activity, enzyme inhibition, and anti‐diabetic effects [13]. In our previous study, we have reported a series of novel thiochroman‐4‐one derivatives containing a semicarbazone moiety with potential α‐glucosidase activity [14]. Thiosemicarbazide derivatives are well known for strong radical scavenging ability, favorable metal chelating performance, and good biocompatibility, and are widely used as pharmacophores to enhance the bioactivity and druggability of parent molecules [15, 16].

Modern drug discovery increasingly relies on computational methods in conjunction with experimental investigation [17]. Molecular docking and molecular dynamics (MD) simulation studies facilitate the elucidation of interaction mechanisms between small molecules and biological targets at the molecular level [18, 19, 20]. These in silico approaches support rational drug design and reduce both the time investment and financial cost of early‐stage research [21].

Based on the molecular hybridization strategy and our previous research on bioactive heterocyclic derivatives, we designed and synthesized a series of novel thiochroman‐4‐one derivatives modified with thiosemicarbazide groups. All target compounds were fully characterized, and their in vitro antioxidant, α‐glucosidase inhibitory, and antibacterial activities were systematically evaluated. Molecular docking and 100 ns MD simulations were further performed to explore the binding mechanisms between active compounds and target proteins. This work provides a new strategy for the development of multifunctional agents with antioxidant, α‐glucosidase inhibitory, and antibacterial properties, and offers promising lead compounds for subsequent structural optimization.

## 2 Results and Discussion

### 2.1 Chemistry and Spectral Characterization

As shown in Scheme 1, target compounds **6a–6r** were synthesized via a five‐step modular reaction starting from commercially available substituted thiophenol, including nucleophilic substitution, intramolecular cyclization, oxidation, hydrazidation, and condensation. The overall yields of all products ranged from 50% to 82%. All target compounds were obtained as solid products, and all compounds adopt a *Z*‑configuration for thiosemicarbazone C═N double bond, which is supported by ^1^H NMR chemical shift characteristics of exchangeable NH protons and literature precedent for thiosemicarbazone analogues. The thioamide moiety (‑NH‑C(═S)‑NH‑) preferentially adopts a planar conformation; possible intramolecular hydrogen‑bond interactions within the thiosemicarbazide scaffold may stabilize the *Z*‑configuration and affect molecular recognition with biological targets. The chemical structures of all derivatives were fully confirmed by ^1^H NMR, ^1^ ^3^C NMR, and HRMS. Compound **6m** was selected as the representative for structural elucidation. In the ^1^H NMR spectrum of **6m**, two characteristic exchangeable N─H protons of the thiosemicarbazide moiety appeared as a singlet at δ 10.59 ppm and a doublet of doublets at δ 7.69 ppm. The thiocarbonyl carbon (C═S) signal of thiosemicarbazide was observed at δ 178.61 ppm in the ^1^ ^3^C NMR spectrum, which was consistent with the typical chemical shift of thiosemicarbazide derivatives. The molecular formula of **6m** was further verified by HRMS (ESI): [M+H]^+^ *m/z* 346.04437, consistent with the calculated value for C_13_H_16_ClN_3_O_2_S_2_ ([M+H]^+^ calcd: 346.044522), confirming the molecular weight and elemental composition.

**SCHEME 1:**
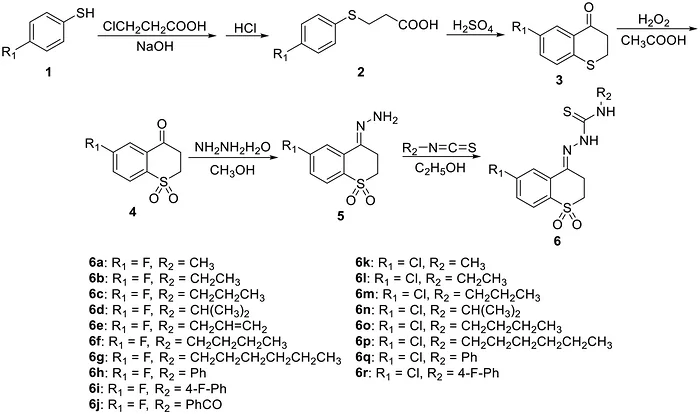
The synthetic route of the target compounds **6a−6r**.

### 2.2 In Vitro Antioxidant and α‐Glucosidase Inhibitory Activity

In vitro antioxidant (DPPH and ABTS radical scavenging) and α‐glucosidase inhibitory activities of compounds **6a**–**6r** were evaluated, with results summarized in Tables 1 and 2. All tested compounds exhibited 100% ABTS radical scavenging activity at the test concentration, indicating excellent in vitro antioxidant potential. Among them, compounds **6a**, **6b**, **6j**, **6k**, and **6m** showed outstanding in vitro ABTS scavenging activity with IC_50_ values of 9.47, 13.76, 13.26, 9.78, and 12.96 µM, respectively, superior to Trolox (18.67 µM). For α‐glucosidase inhibitory activity, compounds **6c**, **6d**, **6f**, **6l**, **6m**, **6n**, and **6o** displayed potent in vitro inhibitory effects with IC_50_ values of 204.29, 235.34, 240.40, 36.71, 27.48, 131.92, and 28.50 µM, respectively, much lower than that of acarbose (367.84 µM). Notably, compound **6m** exhibited excellent dual antioxidant and α‐glucosidase inhibitory activities, which were even better than those of the target compounds reported by our previous study [14].

**TABLE 1:** The in vitro DPPH and ABTS scavenging activities of the target compounds **6a**−**6r**.

Compounds	DPPH scavenging activity (%)	ABTS	
		Scavenging activity (%)	IC_50_ (µM)
**6a**	36.11 ± 1.26	100	9.47 ± 0.83
**6b**	26.94 ± 2.25	100	13.76 ± 3.54
**6c**	22.65 ± 1.62	100	15.51 ± 3.21
**6d**	16.33 ± 0.95	100	17.21 ± 1.97
**6e**	9.99 ± 0.65	100	26.86 ± 3.11
**6f**	18.65 ± 1.14	100	15.87 ± 3.63
**6g**	16.02 ± 1.26	100	16.10 ± 2.28
**6h**	12.35 ± 1.01	100	22.85 ± 4.64
**6i**	15.62 ± 0.95	100	20.26 ± 1.18
**6j**	26.02 ± 1.24	100	13.26 ± 2.68
**6k**	31.92 ± 0.26	100	9.78 ± 3.21
**6l**	25.62 ± 0.54	100	15.33 ± 2.86
**6m**	20.15 ± 0.87	100	12.96 ± 3.03
**6n**	12.36 ± 1.65	100	21.12 ± 3.06
**6o**	5.50 ± 2.21	100	17.47 ± 2.64
**6p**	18.63 ± 1.95	100	17.96 ± 1.68
**6q**	11.90 ± 2.29	100	38.05 ± 5.13
**6r**	16.14 ± 2.24	100	22.81 ± 3.87
Vitamin C	90.20 ± 2.25	/	/
Trolox	/	100	18.67 ± 2.37

**TABLE 2:** The in vitro α‐glucosidase inhibitory activity of the target compounds **6a**−**6r**.

Compounds	Inhibition rate (%)	IC_50_ (µM)
**6a**	24.69 ± 2.01	/
**6b**	30.00 ± 1.26	/
**6c**	82.76 ± 2.65	204.29 ± 6.85
**6d**	67.71 ± 1.94	235.34 ± 5.00
**6e**	48.29 ± 2.22	/
**6f**	69.32 ± 2.10	240.40 ± 6.57
**6g**	22.65 ± 2.24	/
**6h**	31.30 ± 1.94	/
**6i**	37.37 ± 0.95	/
**6j**	30.29 ± 1.11	/
**6k**	43.06 ± 2.65	/
**6l**	88.78 ± 1.95	36.71 ± 4.97
**6m**	90.68 ± 0.56	27.48 ± 2.75
**6n**	73.32 ± 2.29	131.92 ± 4.77
**6o**	89.88 ± 1.26	28.50 ± 3.50
**6p**	36.54 ± 1.65	/
**6q**	39.41 ± 2.29	/
**6r**	34.48 ± 1.65	/
Acarbose	53.00 ± 1.65	367.84 ± 2.42

### 2.3 Structure–Activity Relationship Analysis

For antioxidant ABTS radical‑scavenging activity: most of the fluorine‑substituted and chlorine‑substituted derivatives exhibited excellent ABTS scavenging capacity. Generally, small alkyl substituents (methyl, ethyl, and propyl) on the thiosemicarbazide N‑atom delivered better antioxidant potency than bulky long‑chain alkyl (hexyl) and aromatic phenyl/4‑fluorophenyl substituents. Compound **6a** (*N*‑methyl, F‑substitution) and **6k** (*N*‑methyl, Cl‑substitution) showed the best ABTS scavenging activity among all analogues. Chlorine substitution on the benzene ring tended to produce comparable or slightly stronger antioxidant activity compared with fluorine substitution for identical *N*‑alkyl substituents.

For α‑glucosidase inhibitory activity: chlorine‑substituted series displayed generally stronger inhibitory potency than fluorine‑substituted counterparts. Within chlorine‑substituted derivatives, short‑to‑medium alkyl N‑substituents (ethyl, propyl, and butyl) improved α‑glucosidase inhibition. Compound **6m** (*N*‑propyl, Cl‑substitution) showed optimal α‑glucosidase inhibitory activity. Bulky aromatic *N*‑phenyl or benzamide substituents led to an obvious decrease of α‑glucosidase inhibitory potency.

Compared with our previously reported semicarbazone thiochroman‑4‑one series [14], the current thiosemicarbazide analogues with a C═S thiocarbonyl group achieve improved antioxidant and α‑glucosidase inhibitory potency, confirming that oxygen‑to‑sulfur replacement on the carbonyl pharmacophore can effectively enhance biological activities for this scaffold.

### 2.4 Molecular Docking

Molecular docking was performed to explore the binding mechanism between compound **6m** and two target proteins: human peroxiredoxin 5 (PDB ID: 4K7N; Organism: *Homo sapiens*) and human α‐glucosidase (PDB ID: 3TOP; Organism: *Homo sapiens*). The results suggested that compound **6m** could stably bind to the active pockets of both proteins via multiple non‐covalent interactions, which accounted for its excellent dual bioactivities. In the complex of compound **6m** and peroxiredoxin 5 (Figure 1A), the ligand formed dominant van der Waals interactions with residues ILE119, PHE120, THR44, PRO40, LEU116, and PHE79. Four conventional hydrogen bonds were formed with CYS47, GLY46, and ARG127, and one carbon‐hydrogen bond was observed with THR147. In addition, π‐alkyl and alkyl interactions existed between compound **6m** and PRO45. In the compound **6m** and α‐glucosidase complex (Figure 1B), compound **6m** mainly interacted with TYR1251, ILE1587, THR1586, MET1421, ARG1510, ASP1420, and ASP1157 through van der Waals forces. One conventional hydrogen bond and one carbon‐hydrogen bond were formed with ASP1526. Meanwhile, two π–sulfur interactions with TRP1355 and PHE1560, π–π stacking, and π–alkyl interactions with TRP1369, as well as a π–π stacking interaction with PHE1427, were detected.

**FIGURE 1:**
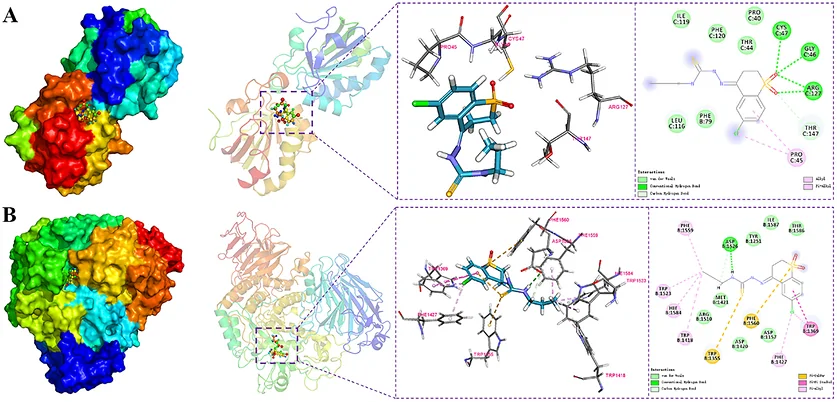
(A) The 3D and 2D molecular binding mode between compound **6m** and the active site of human peroxiredoxin 5 (PDB ID: 4K7N). (B) The 3D and 2D molecular binding mode between compound **6m** and the active site of human α‐glucosidase (PDB ID: 3TOP).

### 2.5 MD Simulation

To comprehensively assess the stability and binding characteristics of the human peroxiredoxin 5–compound **6m** complex, a 100 ns MD simulation was performed. The root‑mean‑square deviation (RMSD, Figure 2A) of the protein backbone and the entire complex gradually converged and remained stable after the initial equilibration phase, suggesting no major conformational rearrangements. The radius of gyration (Rg, Figure 2B) fluctuated within 2.10–2.14 nm throughout the simulation, verifying that the protein maintained a compact, well‑folded tertiary structure. Root‑mean‑square fluctuation (RMSF, Figure 2C) revealed low flexibility for most residues, especially those lining the ligand‑binding pocket, indicating a rigid and stable active site. The interatomic distance between **6m** and the binding‑pocket center remained stable at 1.6–1.8 nm (Figure 2D), and the buried solvent‑accessible surface area (buried SASA, Figure 2E) stayed at 38–40 nm^2^, suggesting firm anchoring of the ligand in the binding cleft (Figure 2F). Free energy landscape and MMPBSA calculations yielded a total binding free energy of −84.090 kJ/mol, with van der Waals interactions as the dominant driving force, complemented by favorable electrostatic and hydrophobic contributions (Figure 2G and Table 3). Per‑residue energy decomposition identified PHE120, LEU116, and PRO40 as key binding residues, with PHE120 contributing the most favorable binding energy (Figure 2H). Hydrogen bond analysis showed the complex maintained an average of 1–2 hydrogen bonds throughout the trajectory (Figure 2I). Collectively, these results suggested that compound **6m** forms a highly stable, energetically favorable complex with human peroxiredoxin 5 (PDB ID: 4K7N).

**FIGURE 2:**
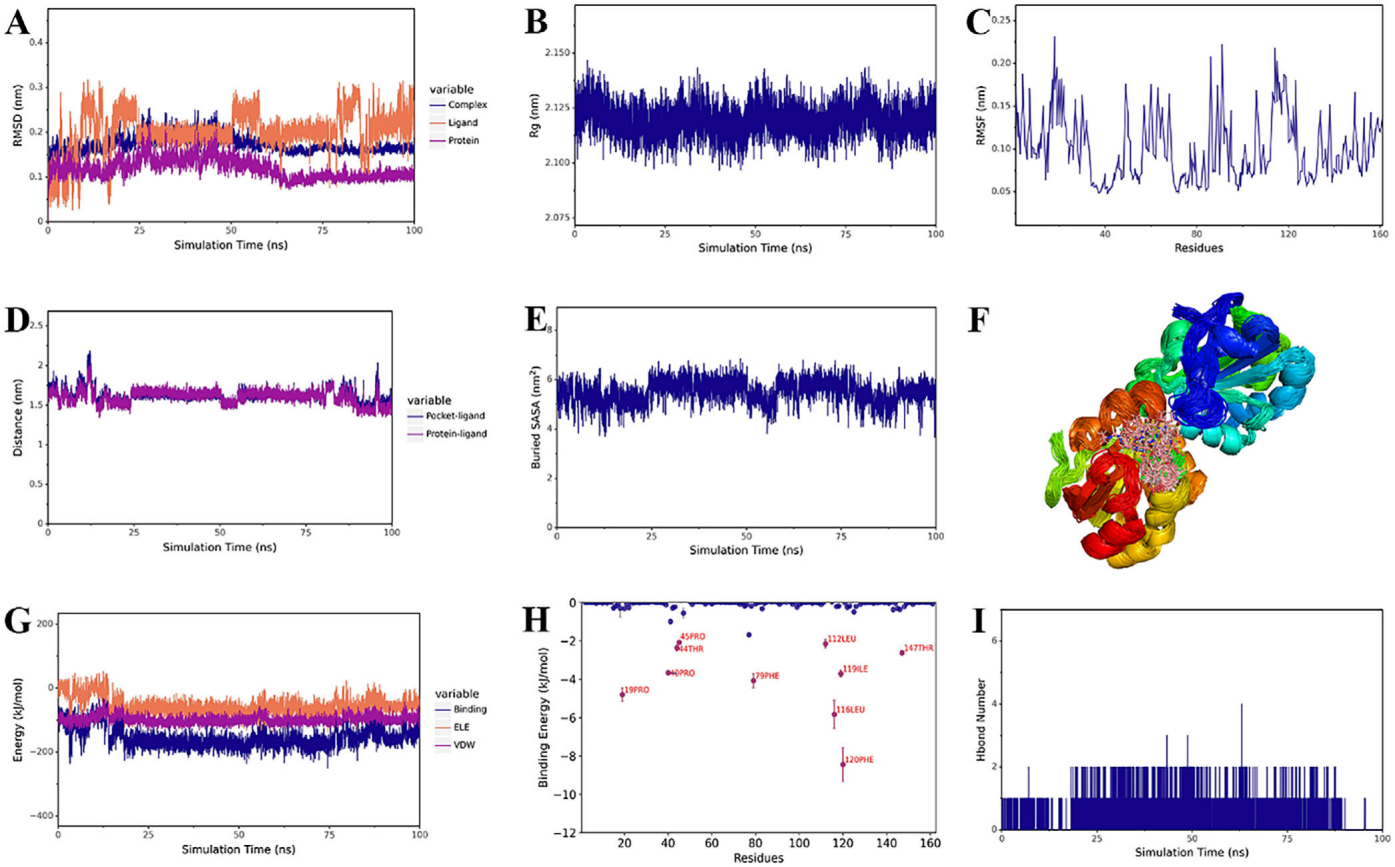
The RMSD (A), Rg (B), RMSF (C), distance (D), buried SASA (E), simulated conformational superposition (F), FEL free energy (G), analysis of residue contribution (H), and hydrogen bond numbers (I) of compound **6m** binding to the active site of human peroxiredoxin 5 (PDB ID: 4K7N).

**TABLE 3:** The binding energy of compound **6m** in stable state when binding to human peroxiredoxin 5 (PDB ID: 4K7N) and human α‐glucosidase (PDB ID: 3TOP), respectively.

Independent components	Energy (kJ/mol)	
	Human peroxiredoxin 5	Human α‐glucosidase
Van der Waals energy (Δ*E*_vdw_)	−105.739 ± 3.084	−90.802 ± 9.252
Electrostatic energy (Δ*E*_ele_)	−10.274 ± 0.629	−13.821 ± 2.598
Polar solvation energy (Δ*E*_pol_)	46.879 ± 1.889	67.634 ± 3.087
Nonpolar solvation energy (Δ*E*_nonpol_)	−14.957 ± 0.132	−14.053 ± 0.88
Total binding energy (Δ*E*_MMPBSA_)	−84.090 ± 1.874	−51.042 ± 8.482
−T∆S	18.768 ± 1.512	25.385 ± 14.339
Total binding free energy (Δ*G*_bind_)	−65.323 ± 3.353	−25.657 ± 17.692

For the human α‐glucosidase–compound **6m** complex, a full 100 ns MD production simulation was carried out. In the initial 0–20 ns, obvious RMSD fluctuations were observed, corresponding to binding‐site structural adjustment and adaptive re‐positioning of compound **6m** within the active pocket. RMSD gradually stabilised within the 20–80 ns simulation window (Figure 3A). Notably, the final 20 ns (80–100 ns) of the trajectory exhibited no substantial RMSD drift; Rg, buried SASA, and hydrogen‐bond occupancy remained steady, and no ligand dissociation or protein partial unfolding events were observed, confirming that the optimised binding pose was sustained until simulation end. Rg values remained stable during 80–100 ns, indicating a compact protein fold (Figure 3B). RMSF analysis revealed a rigid and stable binding‐pocket environment across the final 20 ns (Figure 3C). Distances between ligand and pocket centre stayed consistent, and buried SASA was persistently maintained, supporting firm ligand anchoring (Figure 3D,E). Conformational alignment demonstrated that ligand converged to a dominant binding state throughout 80–100 ns (Figure 3F). The stable 80–100 ns trajectory segment was employed for MMPBSA binding free‐energy calculation. Van der Waals and electrostatic interaction energies remained stable in this window (Figure 3G). Table 3 showed that MMPBSA calculations gave a total binding energy of −51.042 kJ/mol, with van der Waals forces as the main driving force. Per‐residue energy decomposition highlighted HIE1727 and ALA1307 as key residues (Figure 3H). Hydrogen‐bond numbers fluctuated between 0 and 2 during 80–100 ns (Figure 3I), providing persistent polar interactions. These full‐trajectory results suggest stable, energetically plausible binding of compound **6m** to human α‐glucosidase. It should be noted that our biochemical α‐glucosidase inhibition assay used yeast‐derived enzyme, while MD simulation employed human α‐glucosidase crystal structure; this cross‐species difference is an important study limitation.

**FIGURE 3:**
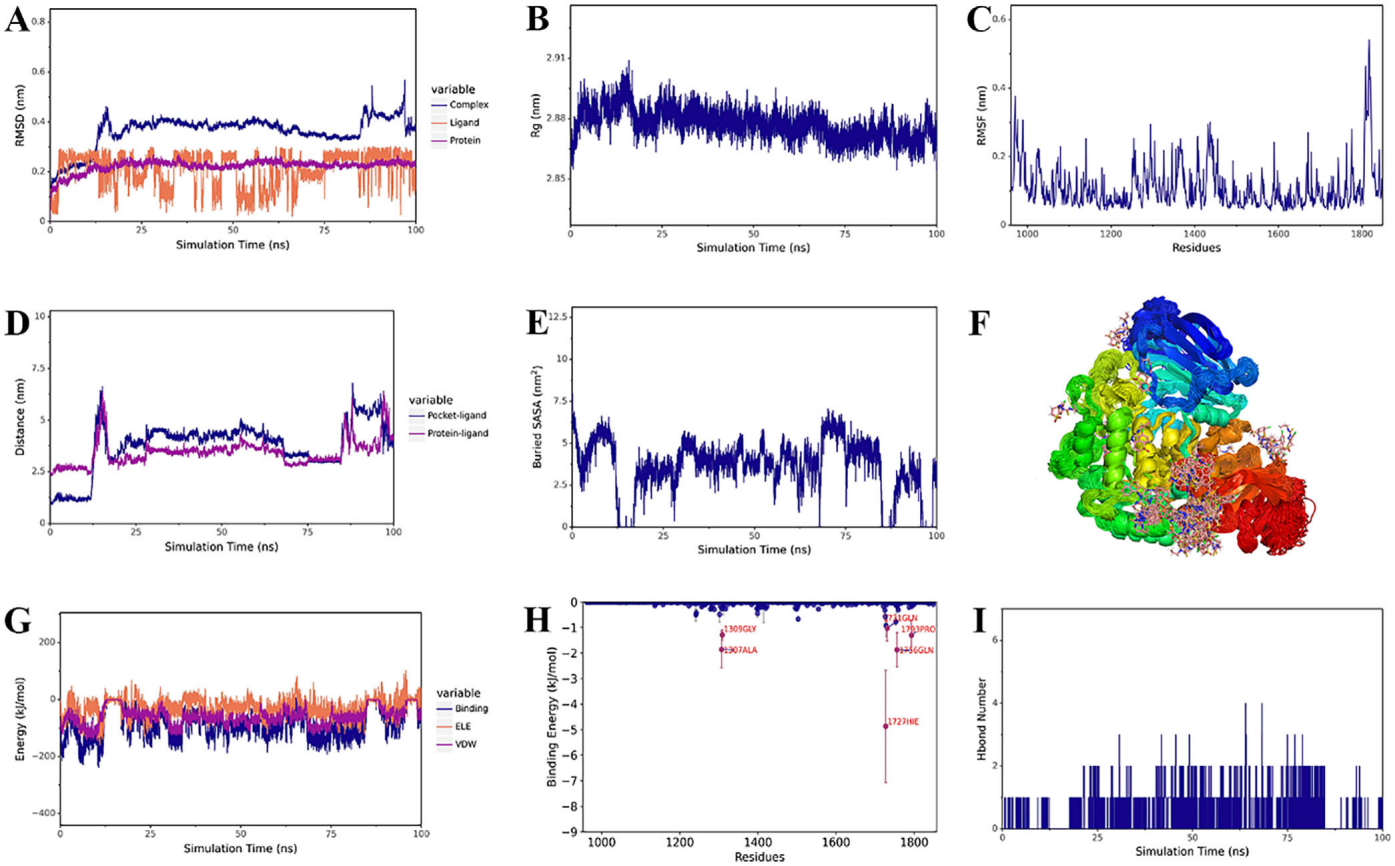
The RMSD (A), Rg (B), RMSF (C), distance (D), buried SASA (E), simulated conformational superposition (F), FEL free energy (G), analysis of residue contribution (H), and hydrogen bond numbers (I) of compound **6m** binding to the active site of human α‐glucosidase (PDB ID: 3TOP).

### 2.6 ADMET and Drug‐Likeness Profiles

As shown in Table S1, in silico ADMET prediction via Deep‑PK suggested compound **6m** possesses favorable absorption, distribution, metabolism, excretion, and safety profiles. Meanwhile, drug‑likeness assessment using Lipinski's Rule of Five showed that compound **6m** fully satisfies all four criteria (molecular weight ≤ 500 Da, logP ≤ 5, hydrogen‑bond donors ≤ 5, hydrogen‑bond acceptors ≤ 10) (Table S2), supporting good oral bioavailability potential. Importantly, the ADMET prediction also alerts potential CYP1A2 and CYP3A4 inhibition risk, together with blood‐‑brain barrier (BBB) penetration possibility. CYP inhibition may lead to potential drug‑drug interaction risk; BBB penetration may result in undesired central nervous system exposure. These predicted liabilities should be considered in future structural optimization. It must be emphasized that all ADMET and druggability results are purely computational predictions, and these are not experimental toxicology or biosafety evidence. No cell or animal toxicity experiments were performed in current study. These results provide a solid foundation for subsequent structural optimization aimed at improving pharmacokinetic behavior while preserving favorable safety.

### 2.7 In Vitro Antibacterial Activity

As summarized in Table 4, compounds **6k** and **6l** demonstrated the most potent in vitro antibacterial activity against *Xoo*, with EC_50_ values of 39.37 and 47.13 µM, respectively—surpassing those of the commercial reference agents bismerthiazol (EC_50_ = 316.23 µM) and thiodiazole copper (EC_50_ = 472.61 µM). Moreover, both compounds exhibited strong in vitro activity against *Xac*, yielding EC_50_ values of 49.21 and 56.17 µM, respectively—again significantly lower than those of bismerthiazol (EC_50_ = 557.28 µM) and thiodiazole copper (EC_50_ = 999.43 µM).

**TABLE 4:** The in vitro antibacterial activities of the target compounds **6a−6r**.

Compounds	Inhibition rate (%)		EC_50_ (µM)	
	*Xoo*	*Xac*	*Xoo*	*Xac*
**6a**	97.65 ± 1.65	92.26 ± 0.95	70.59 ± 2.81	84.92 ± 7.42
**6b**	90.25 ± 2.21	87.65 ± 2.14	91.60 ± 4.81	103.30 ± 4.78
**6c**	87.15 ± 0.68	84.69 ± 1.02	/	/
**6d**	60.25 ± 1.69	52.67 ± 1.95	/	/
**6e**	52.69 ± 2.18	47.95 ± 1.91	/	/
**6f**	50.26 ± 0.85	45.65 ± 1.97	/	/
**6g**	45.65 ± 1.57	42.68 ± 0.85	/	/
**6h**	40.25 ± 1.65	38.65 ± 2.15	/	/
**6i**	43.26 ± 0.84	41.25 ± 1.58	/	/
**6j**	92.54 ± 1.65	90.22 ± 1.68	65.42 ± 3.93	70.30 ± 2.65
**6k**	100 ± 0.15	98.19 ± 1.57	39.37 ± 3.58	49.21 ± 3.84
**6l**	100 ± 0.16	96.54 ± 1.51	47.13 ± 5.69	56.17 ± 4.58
**6m**	94.65 ± 2.14	90.15 ± 1.04	58.40 ± 4.28	87.39 ± 4.57
**6n**	87.41 ± 1.55	82.54 ± 1.25	/	/
**6o**	72.56 ± 1.95	66.54 ± 2.24	/	/
**6p**	65.69 ± 1.87	60.22 ± 1.75	/	/
**6q**	55.21 ± 0.95	51.26 ± 1.54	/	/
**6r**	60.25 ± 1.79	55.15 ± 1.65	/	/
Bismerthiazol	51.29 ± 1.62	32.95 ± 2.69	316.23 ± 4.78	557.28 ± 8.57
Thiodiazole copper	44.86 ± 1.95	22.68 ± 0.23	472.61 ± 7.11	999.43 ± 6.76

## 3 Conclusions

In this study, 18 novel thiochroman‐4‐one derivatives functionalized with a thiosemicarbazide moiety were successfully synthesized, and their antioxidant, α‐glucosidase inhibitory, and antibacterial activities were systematically evaluated. Most of the synthesized compounds displayed strong ABTS radical scavenging activity, and several derivatives exerted remarkable α‐glucosidase inhibitory effects. Compound **6m** was identified as the optimal dual‐active candidate, with an ABTS IC_50_ of 12.96 µM and an α‐glucosidase IC_50_ of 27.48 µM. Its bioactivities were higher than those of Trolox and acarbose, respectively. Molecular docking and 100 ns MD simulations confirmed that compound **6m** could form stable complexes with human peroxiredoxin 5 and human α‐glucosidase mainly via van der Waals interactions. Compound **6k** showed the most prominent antibacterial activity against *Xoo* and *Xac*, with EC_50_ values of 49.21 and 56.17 µM, which were much better than commercial agrochemicals bismerthiazol and thiodiazole copper. By contrast, molecular docking, 100 ns MD simulations, and ADMET/druggability analysis are purely in silico computational predictions, which cannot serve as experimental proof of biological function or target engagement. Molecular docking and MD simulations only suggested plausible hypothetical binding modes between compound **6m** and human peroxiredoxin 5 as well as human α‑glucosidase. These computational observations do not confirm the real biological target of compound **6m**. ADMET and druggability predictions verified the favorable pharmacokinetic properties and biosafety of these compounds. In summary, the thiochroman‐4‐one/thiosemicarbazide hybrid scaffold is a promising structural framework for developing multifunctional agents with antioxidant, hypoglycemic, and antibacterial activities. This work provides valuable lead compounds and a new research direction for the development of novel medicinal and agricultural chemicals.

Several critical limitations of this study warrant explicit acknowledgment. First, the purity of all target compounds used in biological evaluations was assessed by ^1^H NMR and ^1^ ^3^C NMR spectroscopy and confirmed to be ≥ 95%; however, quantitative HPLC‐based purity determinations are not yet available—constituting a methodological constraint. Second, antioxidant activity was evaluated solely via cell‐free chemical assays (DPPH and ABTS radical‐scavenging), with no assessment of endogenous ROS‐modulating effects at the cellular level. Third, α‐glucosidase inhibition was measured using a yeast‐derived enzyme, whereas molecular docking and MD simulations employed the crystal structure of human α‐glucosidase—introducing a species‐level discrepancy between experimental and computational systems. Fourth, the proposed interaction between compound **6m** and human peroxiredoxin 5 rests exclusively on in silico modeling; no biochemical functional validation—such as enzymatic activity modulation or binding affinity measurement—has been conducted. Fifth, ADMET profiling and druggability assessments rely entirely on computational predictions, and cytotoxicity evaluations in normal mammalian cell lines remain unperformed. Sixth, antibacterial activity was tested only against two Gram‐negative phytopathogens (*Xoo* and *Xac*); evaluation against Gram‐positive bacterial strains is necessary to comprehensively define the antimicrobial spectrum. Finally, all bioactivity data are derived from in vitro assays; no cell‐based functional studies, in vivo pharmacological evaluations in animal models, or greenhouse/field‐scale agricultural efficacy trials have been undertaken. Consequently, these compounds remain preclinical candidates and cannot yet be considered validated pharmaceutical or agrochemical agents.

## 4 Experimental Section

### 4.1 General

All reagents and solvents were purchased from commercial suppliers and used without further purification unless otherwise specified. Analytical thin‐layer chromatography (TLC) was performed using pre‐coated silica gel GF_254_ plates. Visualization of chromatographic spots was achieved under ultraviolet (UV) light at 254 nm or via iodine vapor staining. Nuclear magnetic resonance (NMR) spectra were acquired on a 400 MHz NMR spectrometer at ambient temperature. Chemical shifts (*δ*) are reported in parts per million (ppm), with tetramethylsilane (TMS) employed as the internal standard. Coupling constants (*J*) are given in units of hertz (Hz). High‐resolution mass spectrometry (HRMS) analyses were conducted on an electrospray ionization (ESI) time‐of‐flight mass spectrometer.

### 4.2 General Procedure for the Synthesis of the Target Compounds **6a**–**6r**

The target compounds were synthesized following the procedure reported in previous literature [14, 15]. In brief, a mixture of the substituted thiophenol (**1**, 10 mmol) and 3‐chloropropanoic acid (12 mmol) was dissolved in 2 mol/L aqueous sodium hydroxide solution (20 mL). The reaction mixture was stirred at 60°C for 4 h. Upon completion of the reaction, the solution was acidified to pH 2–3 with concentrated hydrochloric acid. The resulting precipitate was collected via filtration, washed with distilled water, and dried to yield intermediate **2**. Under ice‐bath cooling (0°C), intermediate **2** (5 mmol) was slowly added to concentrated sulfuric acid (10 mL). Subsequently, the mixture was heated to 80°C and stirred for 3 h. After cooling to room temperature, the reaction mixture was poured onto crushed ice. The precipitate was collected by filtration, washed with water, and recrystallized from ethanol to afford intermediate **3**. Intermediate **3** (3 mmol) was dissolved in glacial acetic acid (15 mL), followed by the addition of 30% hydrogen peroxide (6 mmol). The mixture was heated to 80°C and stirred overnight. The solvent was evaporated under reduced pressure, and the residue was recrystallized from ethanol to give intermediate **4**. A mixture of intermediate **4** (2 mmol) and 80% hydrazine hydrate (4 mmol) in methanol (20 mL) was heated under reflux for 4 h. After cooling, the precipitate was collected by filtration, washed with cold methanol, and dried to afford intermediate **5**. A solution of intermediate 5 (1 mmol) and the corresponding substituted isothiocyanate (1.2 mmol) in ethanol (15 mL) was refluxed for 4–6 h, with the reaction progress monitored by TLC. After the reaction was completed, the mixture was cooled to room temperature. The solid product was collected by filtration, washed with cold ethanol, and recrystallized from methanol to yield the pure target compounds **6a**–**6r**. The chemical structures of all derivatives were fully confirmed by ^1^H NMR, ^1^ ^3^C NMR, and HRMS. Comprehensive characterization data, including physical properties, ^1^H NMR, ^1^ ^3^C NMR, and HRMS results, are presented below, and full spectral traces (Figures S1–S54) are available in the Supporting Information.

(*Z*)‐2‐(6‐fluoro‐1,1‐dioxidothiochroman‐4‐ylidene)‐*N*‐methylhydrazine‐1‐carbothioamide (**6a**). Light yellow solid, mp. 254.2°C‐255.7°C, yield 81%; ^1^H NMR (400 MHz, DMSO‐*d*_6_, ppm) *δ*: 10.71 (s, 1H, NH), 8.85 (dd, *J* = 12.0, 4.0 Hz, 1H, Ar‐H), 8.45 (dd, *J* = 12.0, 2.0 Hz, 1H, Ar‐H), 7.93 (dd, *J* = 8.0, 4.0 Hz, 1H, Ar‐H), 7.48 (td, *J* = 12.0, 4.0 Hz, 1H, NH), 3.71 (t, *J* = 8.0 Hz, 2H, CH_2_), 3.32 (t, *J* = 8.0 Hz, 2H, CH_2_), 3.09 (d, *J* = 4.0 Hz, 3H, CH_3_); ^13^C NMR (100 MHz, DMSO‐*d*_6_, ppm) *δ*: 179.34, 194.96 (d, *J* = 248.0 Hz), 139.07 (d, *J* = 3.0 Hz), 136.52 (d, *J* = 9.0 Hz), 134.63 (d, *J* = 3.0 Hz), 126.46 (d, *J* = 9.0 Hz), 117.79 (d, *J* = 23.0 Hz), 113.80 (d, *J* = 25.0 Hz), 46.23, 31.76, 25.11; HRMS (ESI) [M+H]^+^ calcd for C_11_H_12_FN_3_O_2_S_2_: 302.042773, found 302.04263.

(*Z*)‐*N*‐ethyl‐2‐(6‐fluoro‐1,1‐dioxidothiochroman‐4‐ylidene)hydrazine‐1‐carbothioamide (**6b**). Light yellow solid, mp. 251.8°C–252.7°C, yield 81%; ^1^H NMR (400 MHz, DMSO‐*d*_6_, ppm) *δ*: 10.62 (s, 1H, NH), 8.94 (t, *J* = 8.0 Hz, 1H, Ar‐H), 8.43 (dd, *J* = 12.0, 4.0 Hz, 1H, Ar‐H), 7.93 (dd, *J* = 8.0, 4.0 Hz, 1H, Ar‐H), 7.49 (td, *J* = 12.0, 4.0 Hz, 1H, NH), 3.70 (t, *J* = 8.0 Hz, 2H, CH_2_), 3.65 (q, *J* = 16.0, 8.0 Hz, 2H, CH_2_CH_3_), 3.31 (t, *J* = 8.0 Hz, 2H, CH_2_), 1.18 (t, *J* = 8.0 Hz, 3H, CH_2_ CH_3_); ^13^C NMR (100 MHz, DMSO‐*d*_6_, ppm) *δ*: 178.31, 164.95 (d, *J* = 248.0 Hz), 139.21 (d, *J* = 2.0 Hz), 136.50 (d, *J* = 9.0 Hz), 134.76 (d, *J* = 3.0 Hz), 126.48 (d, *J* = 10.0 Hz), 117.82 (d, *J* = 24.0 Hz), 113.81 (d, *J* = 24.0 Hz), 46.24, 39.10, 25.13, 14.81; HRMS (ESI) [M+H]^+^ calcd for C_12_H_14_FN_3_O_2_S_2_: 316.058423, found 316.05720.

(*Z*)‐2‐(6‐fluoro‐1,1‐dioxidothiochroman‐4‐ylidene)‐*N*‐propylhydrazine‐1‐carbothioamide (**6c**). Light yellow solid, mp. 221.4°C–223.2°C, yield 80%; ^1^H NMR (400 MHz, DMSO‐*d*_6_, ppm) *δ*: 10.61 (s, 1H, NH), 8.93 (t, *J* = 8.0 Hz, 1H, Ar‐H), 8.43 (dd, *J* = 12.0, 4.0 Hz, 1H, Ar‐H), 7.93 (dd, *J* = 8.0, 4.0 Hz, 1H, Ar‐H), 7.49 (td, *J* = 12.0, 4.0 Hz, 1H, NH), 3.69 (t, *J* = 8.0 Hz, 2H, CH_2_), 3.57 (q, *J* = 16.0, 8.0 Hz, 2H, CH_2_CH_2_CH_3_), 3.31 (t, *J* = 8.0 Hz, 2H, CH_2_), 1.68‐1.58 (m, 2H, CH_2_ CH_2_CH_3_), 0.90 (t, *J* = 8.0 Hz, 3H, CH_2_CH_2_ CH_3_); ^13^C NMR (100 MHz, DMSO‐*d*_6_, ppm) *δ*: 178.59, 164.95 (d, *J* = 248.0 Hz), 139.20 (d, *J* = 2.0 Hz), 136.50 (d, *J* = 9.0 Hz), 134.67 (d, *J* = 2.0 Hz), 126.48 (d, *J* = 10.0 Hz), 117.82 (d, *J* = 24.0 Hz), 113.80 (d, *J* = 24.0 Hz), 46.23, 45.95, 25.13, 22.42, 11.70. HRMS (ESI) [M+H]^+^ calcd for C_13_H_16_FN_3_O_2_S_2_: 330.074073, found 330.07403.

(*Z*)‐2‐(6‐fluoro‐1,1‐dioxidothiochroman‐4‐ylidene)‐*N*‐isopropylhydrazine‐1‐carbothioamide (**6d**). Light yellow solid, mp. 225.4°C–227.2°C, yield 75%; ^1^H NMR (400 MHz, DMSO‐*d*_6_, ppm) *δ*: 10.56 (s, 1H, NH), 8.41 (d, *J* = 8.0 Hz, 1H, Ar‐H), 8.33 (dd, *J* = 12.0, 4.0 Hz, 1H, Ar‐H), 7.94 (dd, *J* = 8.0, 4.0 Hz, 1H, Ar‐H), 7.50 (td, *J* = 12.0, 4.0 Hz, 1H, NH), 4.65‐4.56 (m, 1H, CH), 3.70 (t, *J* = 8.0 Hz, 2H, CH_2_), 3.32 (t, *J* = 8.0 Hz, 2H, CH_2_), 1.27 (d, *J* = 4.0 Hz, 6H, 2 × CH_3_); ^13^C NMR (100 MHz, DMSO‐*d*_6_, ppm) *δ*: 177.47, 164.86 (d, *J* = 248.0 Hz), 139.58 (d, *J* = 3.0 Hz), 136.44 (d, *J* = 9.0 Hz), 134.69 (d, *J* = 3.0 Hz), 126.51 (d, *J* = 9.0 Hz), 117.85 (d, *J* = 24.0 Hz), 113.92 (d, *J* = 25.0 Hz), 46.68, 46.26, 25.19, 22.03. HRMS (ESI) [M+H]^+^ calcd for C_13_H_16_FN_3_O_2_S_2_: 330.074073, found 330.07418.

(*Z*)‐*N*‐allyl‐2‐(6‐fluoro‐1,1‐dioxidothiochroman‐4‐ylidene)hydrazine‐1‐carbothioamide (**6e**). White solid, mp. 216.8°C–218.6°C, yield 60%; ^1^H NMR (400 MHz, DMSO‐*d*_6_, ppm) *δ*: 10.73 (s, 1H, NH), 9.09 (t, *J* = 8.0 Hz, 1H, Ar‐H), 7.47 (dd,, *J* = 12.0, 4.0 Hz, 1H, Ar‐H), 7.93 (dd, *J* = 8.0, 4.0 Hz, 1H, Ar‐H), 7.49 (td, *J* = 12.0, 4.0 Hz, 1H, NH), 5.98‐5.89 (m, 1H, CH), 5.20‐5.11 (m, 2H, CH_2_), 4.29 (t, *J* = 8.0 Hz, 2H, CH_2_), 3.71 (t, *J* = 8.0 Hz, 2H, CH_2_), 3.34 (t, *J* = 8.0 Hz, 2H, CH_2_); ^13^C NMR (100 MHz, DMSO‐*d*_6_, ppm) *δ*: 179.01, 164.96 (d, *J* = 248.0 Hz), 139.46 (d, *J* = 2.0 Hz), 136.47 (d, *J* = 9.0 Hz), 135.20, 134.69 (d, *J* = 2.0 Hz), 117.86 (d, *J* = 10.0 Hz), 115.98, 113.89 (d, *J* = 25.0 Hz), 46.51, 46.25, 25.17; HRMS (ESI) [M+H]^+^ calcd for C_13_H_14_FN_3_O_2_S_2_: 328.058423, found 328.05856.

(*Z*)‐*N*‐butyl‐2‐(6‐fluoro‐1,1‐dioxidothiochroman‐4‐ylidene)hydrazine‐1‐carbothioamide (**6f**). Light yellow solid, mp. 206.6°C–208.2°C, yield 72%; ^1^H NMR (400 MHz, DMSO‐*d*_6_, ppm) *δ*: 10.61 (s, 1H, NH), 8.91 (d, *J* = 8.0 Hz, 1H, Ar‐H), 8.43 (dd, *J* = 12.0, 4.0 Hz, 1H, Ar‐H), 7.93 (dd, *J* = 8.0, 4.0 Hz, 1H, Ar‐H), 7.49 (td, *J* = 12.0, 4.0 Hz, 1H, NH), 3.70 (t, *J* = 8.0 Hz, 2H, CH_2_), 3.62 (q, *J* = 16.0, 8.0 Hz, 2H, CH_2_CH_2_CH_2_CH_3_), 3.31 (t, *J* = 8.0 Hz, 2H, CH_2_), 1.64‐1.57 (m, 2H, CH_2_ CH_2_CH_2_CH_3_), 1.38‐1.29 (m, 2H, CH_2_CH_2_ CH_2_CH_3_), 0.93 (t, *J* = 8.0 Hz, 3H, CH_2_CH_2_CH_2_ CH_3_); ^13^C NMR (100 MHz, DMSO‐*d*_6_, ppm) *δ*: 178.51, 164.95 (d, *J* = 248.0 Hz), 139.17 (d, *J* = 3.0 Hz), 136.51 (d, *J* = 9.0 Hz), 134.67 (d, *J* = 3.0 Hz), 126.58 (d, *J* = 10.0 Hz), 117.81 (d, *J* = 23.0 Hz), 113.80 (d, *J* = 25.0 Hz), 46.23, 44.05, 31.28, 25.13, 20.10, 14.29. HRMS (ESI) [M+H]^+^ calcd for C_14_H_18_FN_3_O_2_S_2_: 344.089723, found 344.09008.

(*Z*)‐2‐(6‐fluoro‐1,1‐dioxidothiochroman‐4‐ylidene)‐*N*‐hexylhydrazine‐1‐carbothioamide (**6g**). White solid, mp. 202.3°C–204.2°C, yield 65%; ^1^H NMR (400 MHz, DMSO‐*d*_6_, ppm) *δ*: 10.61 (s, 1H, NH), 8.91 (d, *J* = 8.0 Hz, 1H, Ar‐H), 8.42 (dd, *J* = 12.0, 4.0 Hz, 1H, Ar‐H), 7.93 (dd, *J* = 8.0, 4.0 Hz, 1H, Ar‐H), 7.49 (td, *J* = 12.0, 4.0 Hz, 1H, NH), 3.70 (t, *J* = 8.0 Hz, 2H, CH_2_), 3.61 (q, *J* = 16.0, 8.0 Hz, 2H, CH_2_CH_2_CH_2_CH_2_CH_2_CH_3_), 3.32 (t, *J* = 8.0 Hz, 2H, CH_2_), 1.63‐1.60 (m, 2H, CH_2_ CH_2_CH_2_CH_2_CH_2_CH_3_), 1.33‐1.30 (m, 6H, CH_2_CH_2_ CH_2_ CH_2_ CH_2_CH_3_), 0.87 (t, *J* = 8.0 Hz, 3H, CH_2_CH_2_CH_2_CH_2_CH_2_ CH_3_); ^13^C NMR (100 MHz, DMSO‐*d*_6_, ppm) *δ*: 178.50, 164.94 (d, *J* = 248.0 Hz), 139.10 (d, *J* = 2.0 Hz), 136.52 (d, *J* = 9.0 Hz), 134.66 (d, *J* = 2.0 Hz), 126.47 (d, *J* = 10.0 Hz), 117.79 (d, *J* = 23.0 Hz), 113.79 (d, *J* = 25.0 Hz), 46.25, 44.32, 31.53, 29.05, 26.53, 25.12, 22.56, 14.37. HRMS (ESI) [M+H]^+^ calcd for C_14_H_18_FN_3_O_2_S_2_: 372.12147, found 372.12147.

(*Z*)‐2‐(6‐fluoro‐1,1‐dioxidothiochroman‐4‐ylidene)‐*N*‐phenylhydrazine‐1‐carbothioamide (**6h**). White solid, mp. 247.3°C–249.2°C, yield 69%; ^1^H NMR (400 MHz, DMSO‐*d*_6_, ppm) *δ*: 11.00 (s, 1H, NH), 10.40 (s, 1H, NH), 8.61 (dd, *J* = 12.0, 4.0 Hz, 1H, Ar‐H) 7.94 (dd, *J* = 8.0, 4.0 Hz, 1H, Ar‐H), 7.52‐7.47 (m, 3H, Ar‐H), 7.42 (t, *J* = 8.0 Hz, 2H, Ar‐H), 7.28 (t, *J* = 8.0 Hz, 1H, Ar‐H), 3.74 (t, *J* = 8.0 Hz, 2H, CH_2_), 3.41 (t, *J* = 8.0 Hz, 2H, CH_2_); ^13^C NMR (100 MHz, DMSO‐*d*_6_, ppm) *δ*: 178.44, 165.0 (d, *J* = 248.0 Hz), 140.38 (d, *J* = 2.0 Hz), 139.57, 136.30 (d, *J* = 9.0 Hz), 134.82 (d, *J* = 2.0 Hz), 128.70, 127.54, 126.53, 126.44 (d, *J* = 10.0 Hz), 118.05 (d, *J* = 23.0 Hz), 114.35 (d, *J* = 25.0 Hz), 46.31, 25.39. HRMS (ESI) [M+H]^+^ calcd for C_16_H_14_FN_3_O_2_S_2_: 364.058423, found 364.05876.

(*Z*)‐2‐(6‐fluoro‐1,1‐dioxidothiochroman‐4‐ylidene)‐*N*‐(4‐fluorophenyl)hydrazine‐1‐carbothioamide (**6i**). Light yellow solid, mp. 244.7°C–246.2°C, yield 62%; ^1^H NMR (400 MHz, DMSO‐*d*_6_, ppm) *δ*: 11.05 (s, 1H, NH), 10.37 (s, 1H, NH), 8.60 (dd, *J* = 12.0, 4.0 Hz, 1H, Ar‐H) 7.95 (dd, *J* = 8.0, 4.0 Hz, 1H, Ar‐H), 7.52‐7.45 (m, 3H, Ar‐H), 7.28‐7.45 (m, 2H, Ar‐H), 3.74 (t, *J* = 8.0 Hz, 2H, CH_2_), 3.40 (t, *J* = 8.0 Hz, 2H, CH_2_); ^13^C NMR (100 MHz, DMSO‐*d*_6_, ppm) *δ*: 178.78, 164.99 (d, *J* = 248.0 Hz), 160.60 (d, *J* = 241.0 Hz), 140.53 (d, *J* = 3.0 Hz), 136.25 (d, *J* = 9.0 Hz), 135.91 (d, *J* = 3.0 Hz), 134.84 (d, *J* = 3.0 Hz), 129.69 (d, *J* = 9.0 Hz), 126.45 (d, *J* = 10.0 Hz), 118.08 (d, *J* = 23.0 Hz), 115.40 (d, *J* = 22.0 Hz), 114.33 (d, *J* = 25.0 Hz), 46.29, 25.40; HRMS (ESI) [M+H]^+^ calcd for C_16_H_13_F_2_N_3_O_2_S_2_: 382.049001, found 382.04891.

(*Z*)‐*N*‐(2‐(6‐fluoro‐1,1‐dioxidothiochroman‐4‐ylidene)hydrazine‐1‐carbonothioyl)benzamide (**6j**). White solid, mp. 217.3°C–219.2°C, yield 60%; ^1^H NMR (400 MHz, DMSO‐*d*_6_, ppm) *δ*: 13.91 (s, 1H, NH), 12.01 (s, 1H, NH), 8.01 (d, *J* = 8.0 Hz, 3H, Ar‐H), 7.94 (dd, *J* = 12.0, 4.0 Hz, 1H, Ar‐H), 7.68 (t, *J* = 8.0 Hz, 1H, Ar‐H), 7.63‐7.54 (m, 3H, Ar‐H), 3.86 (t, *J* = 8.0 Hz, 2H, CH_2_), 3.32 (t, *J* = 8.0 Hz, 2H, CH_2_); ^13^C NMR (100 MHz, DMSO‐*d*_6_, ppm) *δ*: 179.12, 169.18, 164.67 (d, *J* = 249.0 Hz), 147.65, 136.13 (d, *J* = 9.0 Hz), 135.08 (d, *J* = 3.0 Hz), 133.88, 132.16, 129.17 (d, *J* = 36.0 Hz), 126.97 (d, *J* = 9.0 Hz), 118.95 (d, *J* = 22.0 Hz), 113.19 (d, *J* = 24.0 Hz), 46.21, 25.47. HRMS (ESI) [M+H]^+^ calcd for C_17_H_14_FN_3_O_2_S_2_: 392.053337, found 392.05292.

(*Z*)‐2‐(6‐chloro‐1,1‐dioxidothiochroman‐4‐ylidene)‐*N*‐methylhydrazine‐1‐carbothioamide (**6k**). Light yellow solid, mp. 258.3°C–259.7°C, yield 82%; ^1^H NMR (400 MHz, DMSO‐*d*_6_, ppm) *δ*: 10.68 (s, 1H, NH), 8.88 (d, *J* = 8.0 Hz, 1H, Ar‐H), 8.61 (d, *J* = 4.0 Hz, 1H, Ar‐H), 7.86 (d, *J* = 8.0 Hz, 1H, Ar‐H), 7.69 (dd, *J* = 8.0, 4.0 Hz, 1H, NH), 3.71 (t, *J* = 8.0 Hz, 2H, CH_2_), 3.31 (t, *J* = 8.0 Hz, 2H, CH_2_), 3.09 (d, *J* = 4.0 Hz, 3H, CH_3_); ^13^C NMR (100 MHz, DMSO‐*d*_6_, ppm) *δ*: 179.35, 139.12, 138.66, 136.75, 125.21, 130.31, 126.70, 125.31, 46.10, 31.83, 25.08; HRMS (ESI) [M+H]^+^ calcd for C_11_H_12_ClN_3_O_2_S_2_: 318.013222, found 318.01341.

(*Z*)‐2‐(6‐chloro‐1,1‐dioxidothiochroman‐4‐ylidene)‐*N*‐ethylhydrazine‐1‐carbothioamide (**6l**). Light yellow solid, mp. 256.5°C–257.3°C, yield 80%; ^1^H NMR (400 MHz, DMSO‐*d*_6_, ppm) *δ*: 10.59 (s, 1H, NH), 8.95 (t, *J* = 8.0 Hz, 1H, Ar‐H), 8.58 (d, *J* = 4.0 Hz, 1H, Ar‐H), 7.87 (d, *J* = 8.0 Hz, 1H, Ar‐H), 7.69 (dd, *J* = 8.0, 4.0 Hz, 1H, NH), 3.73‐3.64 (m, 4H, CH_2_, CH_2_CH_3_), 3.33 (t, *J* = 8.0 Hz, 2H, CH_2_), 1.19 (t, *J* = 8.0 Hz, 3H, CH_3_); ^13^C NMR (100 MHz, DMSO‐*d*_6_, ppm) *δ*: 178.33, 139.24, 138.65, 136.75, 135.18, 130.32, 126.70, 125.31, 46.14, 39.13, 25.10, 14.81; HRMS (ESI) [M+H]^+^ calcd for C_12_H_14_ClN_3_O_2_S_2_: 332.028872, found 332.02917.

(*Z*)‐2‐(6‐chloro‐1,1‐dioxidothiochroman‐4‐ylidene)‐*N*‐propylhydrazine‐1‐carbothioamide (**6m**). Light yellow solid, mp. 236.3°C–237.6°C, yield 75%; ^1^H NMR (400 MHz, DMSO‐*d*_6_, ppm) *δ*: 10.59 (s, 1H, NH), 8.94 (t, *J* = 8.0 Hz, 1H, Ar‐H), 8.57 (d, *J* = 4.0 Hz, 1H, Ar‐H), 7.87 (d, *J* = 12.0 Hz, 1H, Ar‐H), 7.69 (dd, *J* = 12.0, 4.0 Hz, 1H, NH), 3.71 (t, *J* = 8.0 Hz, 2H, CH_2_), 3.69 (dd, *J* = 16.0, 4.0 Hz, 2H, CH_2_CH_2_CH_3_), 3.33 (t, *J* = 8.0 Hz, 2H, CH_2_), 1.69‐1.60 (m, 2H, CH_2_ CH_2_CH_3_), 0.90 (t, *J* = 8.0 Hz, 3H, CH_2_CH_2_ CH_3_); ^13^C NMR (100 MHz, DMSO‐*d*_6_, ppm) *δ*: 178.61, 139.20, 138.64, 136.76, 135.19, 130.32, 126.69, 125.32, 46.14, 25.10, 22.42, 11.69; HRMS (ESI) [M+H]^+^ calcd for C_13_H_16_ClN_3_O_2_S_2_: 346.044522, found 346.04437.

(*Z*)‐2‐(6‐chloro‐1,1‐dioxidothiochroman‐4‐ylidene)‐*N*‐isopropylhydrazine‐1‐carbothioamide (**6n**). White solid, mp. 248.3°C–249.7°C, yield 70%; ^1^H NMR (400 MHz, DMSO‐*d*_6_, ppm) *δ*: 10.55 (s, 1H, NH), 8.45 (t, *J* = 4.0 Hz, 2H, Ar‐H), 7.87 (d, *J* = 8.0 Hz, 1H, Ar‐H), 7.70 (dd, *J* = 8.0, 4.0 Hz, 1H, NH), 4.62‐4.53 (m, 1H, CH), 3.72 (t, *J* = 8.0 Hz, 2H, CH_2_), 3.33 (t, *J* = 8.0 Hz, 2H, CH_2_), 1.27 (d, *J* 8.0 Hz, 6H, 2 × CH_3_); ^13^C NMR (100 MHz, DMSO‐*d*_6_, ppm) *δ*: 177.54, 139.44, 138.56, 136.79, 135.17, 130.33, 126.82, 125.34, 6.70, 46.18, 25.13, 22.06; HRMS (ESI) [M+H]^+^ calcd for C_13_H_16_ClN_3_O_2_S_2_: 346.044522, found 346.04435.

(*Z*)‐*N*‐butyl‐2‐(6‐chloro‐1,1‐dioxidothiochroman‐4‐ylidene)hydrazine‐1‐carbothioamide (**6o**). Light yellow solid, mp. 224.6°C–226.0°C, yield 65%; ^1^H NMR (400 MHz, DMSO‐*d*_6_, ppm) *δ*: 10.58 (s, 1H, NH), 8.92 (t, *J* = 8.0 Hz, 1H, Ar‐H), 8.57 (d, *J* = 4.0 Hz, 1H, Ar‐H), 7.87 (d, *J* = 12.0 Hz, 1H, Ar‐H), 7.69 (dd, *J* = 8.0, 4.0 Hz, 1H, Ar‐H), 3.71 (t, *J* = 8.0 Hz, 2H, CH_2_), 3.63 (q, *J* = 16.0, 8.0 Hz, 2H, CH_2_CH_2_CH_2_CH_3_), 3.32 (t, *J* = 8.0 Hz, 2H, CH_2_), 1.65‐1.57 (m, 2H, CH_2_ CH_2_CH_2_CH_3_), 1.39‐1.29 (m, 2H, CH_2_CH_2_ CH_2_CH_3_), 0.93 (t, *J* = 8.0 Hz, 3H, CH_2_CH_2_CH_2_ CH_3_); ^13^C NMR (100 MHz, DMSO‐*d*_6_, ppm) *δ*: 178.54, 139.17, 138.63, 136.76, 135.20, 130.31, 126.69, 125.32, 46.13, 44.08, 31.25, 25.10, 20.11, 14.29; HRMS (ESI) [M+H]^+^ calcd for C_14_H_18_ClN_3_O_2_S_2_: 360.060172, found 360.05999.

(*Z*)‐2‐(6‐chloro‐1,1‐dioxidothiochroman‐4‐ylidene)‐*N*‐hexylhydrazine‐1‐carbothioamide (**6p**). Light yellow solid, mp. 242.6°C–244.3°C, yield 60%; ^1^H NMR (400 MHz, DMSO‐*d*_6_, ppm) *δ*: 10.59 (s, 1H, NH), 8.92 (t, *J* = 8.0 Hz, 1H, Ar‐H), 8.56 (d, *J* = 4.0 Hz, 1H, Ar‐H), 7.69 (dd, *J* = 12.0, 4.0 Hz, 1H, NH), 3.71 (t, *J* = 8.0 Hz, 2H, CH_2_), 3.61 (q, *J* = 16.0, 8.0 Hz, 2H, CH_2_CH_2_CH_2_CH_2_CH_2_CH_3_), 3.32 (t, *J* = 8.0 Hz, 2H, CH_2_), 1.64‐1.60 (m, 2H, CH_2_ CH_2_CH_2_CH_2_CH_2_CH_3_), 1.31 (d, *J* = 4.0 Hz, 6H, CH_2_CH_2_ CH_2_ CH_2_ CH_2_CH_3_), 0.88 (t, *J* = 8.0 Hz, 3H, CH_2_CH_2_CH_2_CH_2_CH_2_ CH_3_); ^13^C NMR (100 MHz, DMSO‐*d*_6_, ppm) *δ*: 178.53, 139.14, 138.62, 136.77, 135.21, 130.30, 126.68, 125.32, 46.13, 44.33, 31.52, 29.01, 26.53, 25.09, 22.57, 14.39; HRMS (ESI) [M+H]^+^ calcd for C_16_H_22_ClN_3_O_2_S_2_: 388.091473, found 388.09127.

(*Z*)‐2‐(6‐chloro‐1,1‐dioxidothiochroman‐4‐ylidene)‐*N*‐phenylhydrazine‐1‐carbothioamide (**6q**). Light yellow solid, mp. 252.6°C–254.5°C, yield 58%; ^1^H NMR (400 MHz, DMSO‐*d*_6_, ppm) *δ*: 10.97 (s, 1H, NH), 10.43 (s, 1H, NH), 8.75 (d, *J* = 4.0 Hz, 1H, Ar‐H), 7.88 (d, *J* = 8.0 Hz, 1H, Ar‐H), 7.70 (dd, *J* = 8.0, 4.0 Hz, 1H, Ar‐H), 7.49 (d, *J* = 8.0 Hz, 2H, Ar‐H), 7.42 (t, *J* = 8.0 Hz, 2H, Ar‐H), 7.28 (t, *J* = 8.0 Hz, 1H, Ar‐H), 3.75 (t, *J* = 8.0 Hz, 2H, CH_2_), 3.41 (t, *J* = 8.0 Hz, 2H, CH_2_); ^13^C NMR (100 MHz, DMSO‐*d*_6_, ppm) *δ*: 178.45, 140.34, 139.61, 138.70, 136.92, 134.98, 130.56, 128.73, 127.54, 127.17, 126.56, 125.29, 46.19, 25.35; HRMS (ESI) [M+H]^+^ calcd for C_16_H_14_ClN_3_O_2_S_2_: 380.028872, found 380.02853.

(*Z*)‐2‐(6‐chloro‐1,1‐dioxidothiochroman‐4‐ylidene)‐*N*‐(4‐fluorophenyl)hydrazine‐1‐carbothioamide (**6r**). White solid, mp. 251.6°C–253.6, yield 50%; ^1^H NMR (400 MHz, DMSO‐*d*_6_, ppm) *δ*: 11.03 (s, 1H, NH), 10.41 (s, 1H, NH), 8.75 (d, *J* = 4.0 Hz, 1H, Ar‐H), 7.89 (d, *J* = 12.0 Hz, 1H, Ar‐H), 7.70 (dd, *J* = 8.0, 4.0 Hz, 1H, Ar‐H), 7.50 (dd, *J* = 8.0, 4.0 Hz, 2H, Ar‐H), 7.25 (t, *J* = 8.0 Hz, 2H, Ar‐H), 3.76 (t, *J* = 8.0 Hz, 2H, CH_2_), 3.41 (t, *J* = 8.0 Hz, 2H, CH_2_); ^13^C NMR (100 MHz, DMSO‐*d*_6_, ppm) *δ*: 178.80, 160.61 (d, *J* = 242.0 Hz), 140.52, 138.70, 136.95, 134.94, 130.60, 129.71 (d, *J* = 9.0 Hz), 127.15, 125.30, 115.43 (d, *J* = 22.0 Hz), 46.17, 25.36; HRMS (ESI) [M+H]^+^ calcd for C_16_H_13_ClFN_3_O_2_S_2_: 398.019451, found 398.01891.

### 4.3 In Vitro Antioxidant and α‐Glucosidase Inhibitory Activity Assay

The in vitro antioxidant activities (including DPPH and ABTS radical scavenging capacity) and α‐glucosidase inhibitory activities of the synthesized compounds **6a**−**6r** were evaluated in accordance with the protocols established in our previous work [16, 22, 23]. The specific experimental procedures are described as follows:

For the DPPH radical scavenging assay: A stock solution of each test compound was prepared by dissolving 5.0 mg of the target compound in 100 mL of methanol, obtaining a final concentration of 50 µg/mL. Next, 160 µL of the compound stock solution was mixed with 40 µL of 0.5 mM DPPH methanolic solution in a 96‐well microplate, resulting in a final test concentration of 40 µg/mL. After 30 min of incubation in the dark at room temperature, the absorbance was measured at 517 nm using a Multiskan Sky full‐spectrum microplate spectrophotometer (Thermo Fisher Scientific, Shanghai, China). DMSO was used as the blank (negative control), while Vitamin C was adopted as the positive reference antioxidant.

For the ABTS radical scavenging assay: ABTS stock solution was prepared by mixing equal volumes (0.2 mL each) of 2.6 mM K_2_S_2_O_8_ solution and 7.4 mM ABTS solution, followed by 12 h of incubation in the dark at room temperature. The resulting deep‐blue ABTS radical solution was then diluted 50‐fold with PBS (pH 7.4) to prepare the working reagent. A primary stock solution of each test compound was prepared by dissolving 6.25 mg of the target compound in 100 mL of methanol to reach a concentration of 62.5 µg/mL. For the assay, 160 µL of the compound stock solution was mixed with 40 µL of the diluted ABTS working reagent in a 96‐well microplate, giving a final compound concentration of 50 µg/mL. After 30 min of reaction in the dark, the absorbance was recorded at 734 nm using the Multiskan Sky full‐spectrum microplate spectrophotometer (Thermo Fisher Scientific, Shanghai, China). DMSO was used as the blank control, and Trolox was adopted as the reference antioxidant.

For the α‐glucosidase inhibitory activity assay: 4 mg of each test compound was dissolved in 100 µL of DMSO, then diluted with 900 µL of PBS (pH 6.8) to prepare a compound stock solution with a concentration of 4000 µg/mL. In a 96‐well microplate, reagents were added sequentially as follows: 20 µL of α‐glucosidase (final concentration 0.5 U/mL, Aladdin, China), 70 µL of PBS, 80 µL of 0.2 M Na_2_CO_3_, and 10 µL of the test compound solution. After 10 min of pre‐incubation at 37°C, the reaction was initiated by adding 20 µL of PNPG (final concentration 2.5 mM), followed by another 30 min of incubation at 37°C. The absorbance was measured at 405 nm using the Multiskan Sky full‐spectrum microplate spectrophotometer (Thermo Fisher Scientific, Shanghai, China) to determine enzyme inhibitory activity. DMSO was used as the negative control, while acarbose was adopted as the reference inhibitor.

### 4.4 Molecular Docking and MD Simulations

Human peroxiredoxin 5 is a ubiquitous, atypical 2‐Cys peroxiredoxin that catalyzes the reduction of H_2_O_2_, alkyl hydroperoxides, and peroxynitrite, which are the major intracellular ROS responsible for oxidative stress. It is a well‐characterized antioxidant enzyme that has been established as a relevant molecular target for evaluating antioxidant agents [24]. Human intestinal α‐glucosidase, an enzyme that catalyzes the breakdown of carbohydrates into glucose, plays a crucial role in mediating the digestion of dietary polysaccharides and modulating post‐meal blood glucose levels [25]. Molecular docking and 100 ns MD simulations are key in silico techniques that are widely applied to characterize the binding interactions between bioactive small molecules and biological macromolecular targets [26]. To elucidate the binding mode and key interaction forces between the potent derivative **6m** and these enzymes, we performed molecular docking simulations using AutoDock Tools 1.5.6, following our previously established protocol [15]. The three‐dimensional crystal structures of human peroxiredoxin 5 and human intestinal α‐glucosidase—sourced from the RCSB Protein Data Bank (https://www.rcsb.org/)—were utilized as receptor models in molecular docking analyses to assess plausible binding modes. The 2D structure of the target compound was first drawn in ChemDraw Ultra 12.0 and subsequently converted into a 3D SDF file; this was then transformed into PDB format using Open Babel. Prior to docking, protein structures were prepared with AutoDock Tools 1.5.6: water molecules were eliminated, polar hydrogen atoms were added, and partial atomic charges were assigned via the Gasteiger method. Both the ligand and protein structures were then formatted into PDBQT files to ensure compatibility with the docking engine.

Docking simulations were carried out using AutoDock 4.2 (integrated within AutoDock Tools 1.5.6), and resultant binding poses were evaluated based on predicted binding affinity and structural clustering behavior. A cubic grid box centered at the geometric centroid of the human peroxiredoxin 5 and human intestinal α‐glucosidase catalytic cavity was generated, with protomol_bloat = 0 and protomol_threshold = 0.50 Å; all other grid and genetic algorithm parameters retained default AutoDock settings. The Lamarckian genetic algorithm was applied for conformational sampling, with 10 independent docking runs performed for each ligand. The top 10 clustered binding poses were retained, and the optimal conformation was selected based on two criteria: highest consensus C‐score and high spatial overlap with the co‐crystallized ligand binding region [30]. Parallel Surflex‐Dock simulations were run under rigid receptor + fully flexible ligand conditions for cross‐verification. Post‐docking interaction visualization was conducted via PyMOL 2.1 (3D complex) and LigPlot^+^ 2.3.1 (2D residue contact diagram) to annotate hydrogen bonds, π‐alkyl stacking, and hydrophobic van der Waals contacts.

The protein's force field parameters and topological structure were assigned using the AMBER99SB‐ILDN variant, whereas ligand molecules were modeled with the Generalized Amber force field (GAFF). To accommodate the solvated system, a cubic water box was generated with a minimum protein–box edge distance of at least 1.0 nm. Solvation was performed using the SPC216 water model; subsequently, selected water molecules were substituted with Na^+^ and Cl^−^ counterions to neutralize the total system charge. Initial structural relaxation was achieved via steepest descent energy minimization to alleviate unfavorable atomic contacts and geometric strain. Equilibration proceeded in two sequential phases: first, a 100 ps NVT ensemble simulation at 300 K, utilizing velocity‐rescaling for temperature regulation; second, a 100 ps NPT ensemble run at the same temperature (300 K) and pressure (1 bar), with pressure maintained via the Parrinello–Rahman barostat. Following equilibration, an unrestrained 100 ns MD production run was executed under periodic boundary conditions. Numerical integration of the equations of motion employed the leapfrog integrator with a 2‐fs timestep. Long‐range electrostatic interactions were handled using the particle mesh Ewald (PME) method, configured with a Fourier grid spacing of 0.16 nm; all covalent bonds—including those involving hydrogen atoms—were constrained using the LINCS algorithm. Trajectory inspection, quantitative analysis, and molecular visualization were carried out using VMD (Version 1.9.3) and PyMOL (Version 2.4.1). Structural dynamics and energetic profiling leveraged Desmond's native analysis tools. Free energy landscapes (FELs) were generated by mapping MD trajectory frames onto the leading two principal components (PC1 and PC2) derived from covariance‐based principal component analysis (PCA). The compound's binding free energy was estimated using the gmx_mmpbsa tool (http://jerkwin.github.io/gmxtool) [26]. Specifically, Δ*E*_ele_ denotes the electrostatic contribution; Δ*E*_vdw_ accounts for van der Waals interactions; Δ*E*_pol_ reflects the polar component of solvation energy—often approximated via electrostatic potential calculations; and Δ*E*_nonpol_ captures the nonpolar solvation contribution, commonly associated with hydrophobic effects. The MM/PBSA energy term, Δ*E*_MMPBSA_, was calculated as the algebraic sum of these four components (Δ*E*_ele_ + Δ*E*_vdw_ + Δ*E*_pol_ + Δ*E*_nonpol_). The Gibbs binding energy, Δ*G*_bind_, was subsequently obtained by adding the entropic contribution (−TΔS) to Δ*E*_MMPBSA_. Given that the calculation of ‐TΔS entails substantial error, this term is frequently excluded in binding energy comparisons, and Δ*E*_MMPBSA_ can be directly employed as the binding energy. All figures and graphical representations were generated using Origin 2021 for data processing and visualization.

### 4.5 ADMET and Drug‐Likeness Analysis

The ADMET (absorption, distribution, metabolism, excretion, toxicity) profile and drug‐like properties of compound **6m** were assessed on November 11, 2025, using the Deep‐PK platform (https://biosig.lab.uq.edu.au/deeppk/) and SwissADME tool (https://www.swissadme.ch/), respectively [23].

### 4.6 In Vitro Antibacterial Activity Assay

To investigate the potential application of the target compounds in agricultural disease management, the in vitro antibacterial activities of compounds **6a**−**6r** against *Xoo* (strain PXO99A) and *Xac* (strain 29‑1) belong to Gram‑negative plant‑pathogenic bacteria, were evaluated via a previously established protocol [27]. Briefly, 7.5 mg of each target compound was dissolved in 150 µL of DMSO. Subsequently, 40 µL of the resulting stock solution was transferred separately into two 15 mL centrifuge tubes, each containing 4 mL of 0.1% Tween aqueous solution. Next, 1 mL of each diluted solution was added into triplicate test tubes containing 4 mL of nutrient broth (NB) medium, yielding final test solutions at concentrations of 100 µg/mL, respectively. Afterwards, 40 µL of NB medium pre‐inoculated with *Xoo* or *Xac* was added to each test tube. All inoculated test tubes were incubated at 30°C with shaking at 180 rpm for 24–48 h, until the optical density at 595 nm (OD_595_) of the bacterial culture reached 0.6–0.8. DMSO was set as the negative control, and the inhibition rate I (%) was calculated following the method reported in the previous literature [27].

### 4.7 Data Analysis

The in vitro scavenging activities against DPPH and ABTS free radicals, as well as the inhibitory effect on α‐glucosidase, were calculated and expressed as mean values ± standard deviation (SD) using the specified formula. In parallel, IC_50_ or EC_50_ values, representing the concentration required for 50% inhibition, were derived through GraphPad Prism 5 software (San Diego, CA, USA). All experiments were performed in triplicate (*n* = 3). Data are presented as mean ± SD.

$$\text{Inhibition rate}\;\left(\%\right)=\frac{\left({\text{OD}}_{\text{control}}-{\text{OD}}_{\text{sample}}\right)}{{\text{OD}}_{\text{control}}}\times 100\%$$

## Author Contributions

**Wenmin Pan**: conceptualization, methodology, software, validation, resources, writing – original draft preparation. **You Zhang**: validation, formal analysis, investigation, data curation, **Xiang Wang**: writing – review and editing, project administration, funding acquisition. **Pei Li**: writing – review and editing, project administration, funding acquisition. All authors have read and agreed to the published version of the manuscript.

## Conflicts of Interest

The authors declare no conflicts of interest.

## Supporting information



Supporting information for this article is available on the WWW under https://doi.org/10.1002/MS‐number. Table S1: Predicted ADMET parameters of compound **6m**. Table S2: Drug‐likeness parameters of compound **6m**. Figures S1–S54: The ^1^H NMR, ^13^C NMR, and HRMS spectral of compounds **6a**−**6r**.
**Supporting File**: cbdv71927‐sup‐0001‐SuppMat.docx

## Data Availability

The data that support the findings of this study are available from the corresponding author upon reasonable request.
