# Supplementary material for: Novel Thiochroman‐4‐one Derivatives Bearing a Thiosemicarbazide Scaffold With Potential Antioxidant, α‐Glucosidase Inhibitory, and Antibacterial Activities
Source: Chem Biodivers. 2026 Nov 14;23(11):e71927. doi: 10.1002/cbdv.71927 (PMC13769025; doi:10.1002/cbdv.71927)
Supplement: Supplementary file 1 [file CBDV-23-e71927-s001.docx]

**Table S1**. Predicted ADMET parameters of compound **6m**.

| ADMET parameters | ADMET name | Physicochemical properties |
| --- | --- | --- |
| Absorption | Human oral bioavailability 20% | Bioavailable |
|  | Human intestinal absorption | Absorbed |
|  | Human oral bioavailability 50% | Bioavailable |
|  | p-Glycoprotein inhibitor | Inhibitor |
|  | p-Glycoprotein substrate | Non-substrate |
|  | Skin permeability | Low skin permeability: log KP > -2.5 |
| Distribution | Blood-brain barrier | Penetrable |
| Metabolism | CYP 1A2 inhibitor | Inhibitor |
|  | CYP 1A2 substrate | Substrate |
|  | CYP 2C19 inhibitor | Non-inhibitor |
|  | CYP 2C19 substrate | Substrate |
|  | CYP 2C9 inhibitor | Non-inhibitor |
|  | CYP 2C9 substrate | Substrate |
|  | CYP 2D6 inhibitor | Non-inhibitor |
|  | CYP 2D6 substrate | Non-substrate |
|  | CYP 3A4 inhibitor | Inhibitor |
|  | CYP 3A4 substrate | Non-substrate |
|  | OATP1B1 | Non-inhibitor |
|  | OATP1B3 | Non-inhibitor |
| Excretion | Organic cation transporter 2 | Non-inhibitor |
|  | Half-life of drug | < 3hs |
| Toxicity | AMES Mutagenesis | Safe |
|  | Avian | Safe |
|  | Biodegradation | Safe |
|  | Carcinogenesis | Safe |
|  | Eye corrosion | Safe |
|  | Eye irritation | Safe |
|  | hERG blockers | Safe |
|  | NR-AhR | Safe |
|  | NR-AR | Safe |
|  | NR-AR-LBD | Safe |
|  | NR-aromatase | Safe |
|  | NR-ER | Safe |
|  | NR-ER-LBD | Safe |
|  | NR-GR | Safe |
|  | NR-PPAR-gamma | Safe |
|  | NR-TR | Safe |
|  | Skin sensitisation | Safe |
|  | SR-ATAD5 | Safe |
|  | SR-HSE | Safe |
|  | SR-MMP | Safe |
|  | SR-p53 | Safe |

**Table S2**. Drug-likeness parameters of compound **6m**.

| Drug-likeness parameters | Physicochemical properties |
| --- | --- |
| Molecular mass (MW) | 345.04 |
| logP | 2.63 |
| Hydrogen bond donors | 2 |
| Hydrogen bond acceptors | 3 |

**Figure S1.** ^1^H NMR spectra of compound **6a**

**Figure S2.** ^13^C NMR spectra of compound **6a**

**Figure S3.** HRMS spectra of compound **6a**

**Figure S4.** ^1^H NMR spectra of compound **6b**

**Figure S5.** ^13^C NMR spectra of compound **6b**

**Figure S6.** HRMS spectra of compound **6b**

**Figure S7.** ^1^H NMR spectra of compound **6c**

**Figure S8.** ^13^C NMR spectra of compound **6c**

**Figure S9.** HRMS spectra of compound **6c**

**Figure S10.** ^1^H NMR spectra of compound **6d**

**Figure S11.** ^13^C NMR spectra of compound **6d**

**Figure S12.** HRMS spectra of compound **6d**

**Figure S13.** ^1^H NMR spectra of compound **6e**

**Figure S14.** ^13^C NMR spectra of compound **6e**

**Figure S15.** HRMS spectra of compound **6e**

**Figure S16.** ^1^H NMR spectra of compound **6f**

**Figure S17.** ^13^C NMR spectra of compound **6f**

**Figure S18.** HRMS spectra of compound **6f**

**Figure S19.** ^1^H NMR spectra of compound **6g**

**Figure S20.** ^13^C NMR spectra of compound **6g**

**Figure S21.** HRMS spectra of compound **6g**

**Figure S22.** ^1^H NMR spectra of compound **6h**

**Figure S23.** ^13^C NMR spectra of compound **6h**

**Figure S24.** HRMS spectra of compound **6h**

**Figure S25.** ^1^H NMR spectra of compound **6i**

**Figure S26.** ^13^C NMR spectra of compound **6i**

**Figure S27.** HRMS spectra of compound **6i**

**Figure S28.** ^1^H NMR spectra of compound **6j**

**Figure S29.** ^13^C NMR spectra of compound **6j**

**Figure S30.** HRMS spectra of compound **6j**

**Figure S31.** ^1^H NMR spectra of compound **6k**

**Figure S32.** ^13^C NMR spectra of compound **6k**

**Figure S33.** HRMS spectra of compound **6k**

**Figure S34.** ^1^H NMR spectra of compound **6l**

**Figure S35.** ^13^C NMR spectra of compound **6l**

**Figure S36.** HRMS spectra of compound **6l**

**Figure S37.** ^1^H NMR spectra of compound **6m**

**Figure S38.** ^13^C NMR spectra of compound **6m**

**Figure S39.** HRMS spectra of compound **6m**

**Figure S40.** ^1^H NMR spectra of compound **6n**

**Figure S41.** ^13^C NMR spectra of compound **6n**

**Figure S42.** HRMS spectra of compound **6n**

**Figure S43.** ^1^H NMR spectra of compound **6o**

**Figure S44.** ^13^C NMR spectra of compound **6o**

**Figure S45.** HRMS spectra of compound **6o**

**Figure S46.** ^1^H NMR spectra of compound **6p**

**Figure S47.** ^13^C NMR spectra of compound **6p**

**Figure S48.** HRMS spectra of compound **6p**

**Figure S49.** ^1^H NMR spectra of compound **6q**

**Figure S50.** ^13^C NMR spectra of compound **6q**

**Figure S51.** HRMS spectra of compound **6q**

**Figure S52.** ^1^H NMR spectra of compound **6r**

**Figure S53.** ^13^C NMR spectra of compound **6r**

**Figure S54.** HRMS spectra of compound **6r**
